# Data on plug-based large-bore arteriotomy vascular closure device related access complications

**DOI:** 10.1016/j.dib.2021.106969

**Published:** 2021-03-18

**Authors:** Rutger-Jan Nuis, David Wood, Herbert Kroon, Maarten van Wiechen, Darra Bigelow, Chris Buller, Joost Daemen, Peter de Jaegere, Zvonimir Krajcer, John Webb, Nicolas Van Mieghem

**Affiliations:** a Department of Cardiology, Erasmus Medical Center, Room Nt-645, Dr. Molewaterplein 40, 3015 GD Rotterdam, the Netherlands; b Centre for Cardiovascular and Heart Valve Innovation, St. Paul's and Vancouver General Hospital, Vancouver, Canada; c Clinical and Medical Affairs, Teleflex Inc., Exton, PA, USA; d Baylor St Luke Hospital, Houston, TX, USA

**Keywords:** TAVI, Vascular complications, Closure device, Access site, Aortic stenosis

## Abstract

This article provides supplementary tables and figures to the research article: Frequency, Impact and Predictors of Access Complications with Plug-Based Large-Bore Arteriotomy Closure - A patient level meta-analysis [1]. The data provide insight in the type and management of access complications related to the plug-based MANTA vascular closure device (VCD) for large-bore catheter-based cardiovascular interventions. Since MANTA is mostly used in transcatheter aortic valve replacement (TAVR) procedures, this article also contains a sub-group analysis on TAVR procedures using contemporary valve-platforms. Further, data describing MANTA hemostasis times and mortality causes are included. For this dataset, individual patient data were derived from a European and a North American device approval study (the Conformite Européene [CE] mark study and the investigational device exemption SAFE-MANTA study [2,3]) in addition to a post-approval registry (the MARVEL registry [4]) covering a total of 891 patients who were enrolled between 2015 and 2019 across 28 investigational sites. Eligibility criteria were most stringent in the SAFE MANTA study (38% of patients) whereas the MARVEL registry applied liberal and only relative exclusion criteria (56% of patients). A total of 78 Roll-in cases (i.e. first or second time operator use of the MANTA VCD) who were excluded from analysis in SAFE MANTA were included in the present to evaluate a potential learning curve effect. Therefore, this dataset reflects the largest study population undergoing arteriotomy closure with the MANTA VCD by operators at various levels of experience, which can be valuable to further build on research regarding percutaneous large-bore arteriotomy management.

## Specifications Table

**Table:** 

Subject	Cardiology and Cardiovascular Medicine
Specific subject area	Large-bore catheter-based cardiac and vascular interventions
Type of data	Tables and Figures
How data were acquired	In this patient-level meta-analysis, data were derived from two multicenter, prospective, single arm medical device approval studies (the CE mark study [2], and the Investigational Device Exemption SAFE-MANTA Pivotal Study [3]) in addition to a multicenter prospective post-approval study (the MAnta Registry for Vascular Large-borE CLosure [MARVEL] registry [4]). Statistical analyses were performed using Statistical Package for the Social Sciences version 25 (IBM, Armonk, New York)
Data format	Analysed
Parameters for data collection	The one inclusion criterion in all studies was: all patients undergoing percutaneous cardiac interventions with large-bore catheter sizes and planned access closure using the MANTA VCD.Exclusion criteria in each of the three studies are detailed in Table 1. The main exclusion criteria were: - Morbid obesity or cachexia (body mass index >40 or <20 kg/m^2^) - Excessive femoral calcium or severe peripheral vascular disease - Marked tortuosity of the iliofemoral tract - Puncture site other than the common femoral artery
Description of data collection	All clinical data were prospectively collected and clinical follow-up was planned between 30- and 60 days after the procedure. An independent clinical research organization overlooked study conduction and monitoring. All vascular- and bleeding complications were adjudicated by independent clinical event committees. For the purpose of this patient-level meta-analysis, a selection of individual patient data were merged in a dedicated database and used for these analyses.
Data source location	Source location of CE-mark and SAFE-MANTA trial data: Teleflex Inc. Exton Pennsylvania United States of America Source location of MARVEL trial data: Erasmus Medical Center Rotterdam The Netherlands
Data accessibility	With the article
Related research article	Frequency, Impact and Predictors of Access Complications with Plug-Based Large-Bore Arteriotomy Closure - A patient level meta-analysis. RJ Nuis, D Wood, H Kroon, M van Wiechen, D Bigelow, C Buller, J Daemen, P de Jaegere, Z Krajcer, J Webb, N Van Mieghem. Cardiovascular Revascularization Medicine. 2021. https://doi.org/10.1016/j.carrev.2021.02.017

## Value of the Data

• Vascular management in large-bore catheter-based interventions is challenging and affects patient outcome. These supplementary data provide detailed insight into the type and management of MANTA related access complications across various large-bore catheter-based interventions and also in a more homogenous population of patients undergoing TAVR using contemporary valves.
• The patients in this dataset reflect the largest study population undergoing arteriotomy closure with the MANTA VCD by operators at various levels of experience. It can be valuable to further build on research regarding large-bore arteriotomy management which ultimately benefits patients undergoing large-caliber catheter-based interventions.
• The data described should help understand the mechanisms of MANTA related access complications in patients undergoing various catheter-based interventions such as TAVR, which can be useful to optimize risk stratification, pre-procedural planning, vascular management and future iterations in (plug-based) closure technologies.

## 1 Data Description

**Fig. 1:**
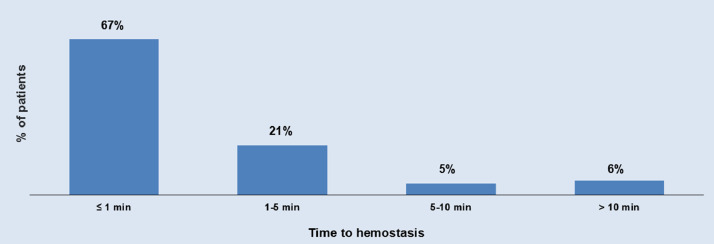
Distribution of hemostasis times.

**Fig. 2:**
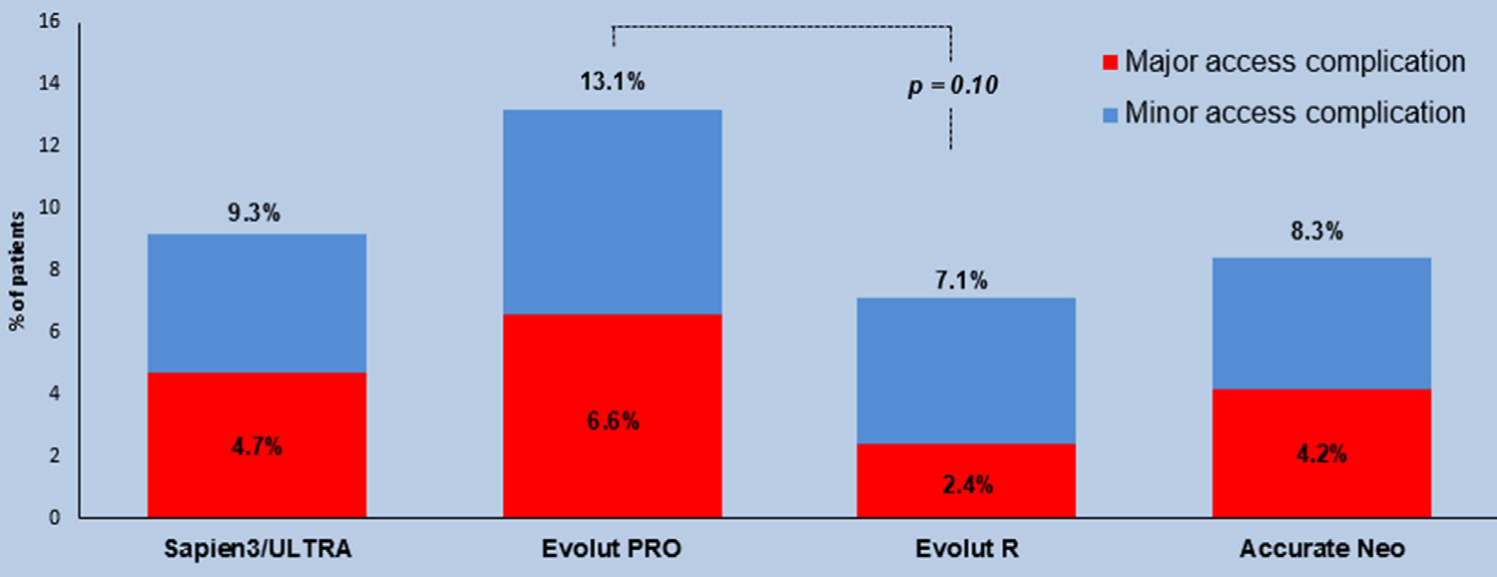
Frequency of major and minor access complications per valve-platform.

This dataset provides relevant details on the frequency, impact and predictors of MANTA related access complications. Data are presented in Tables and Figures. Table 1 describes the general characteristics of each of the three studies from which data were used for the present dataset. Each study had a prospective, observational, multicenter design with similar inclusion criteria but various exclusion criteria. Table 2 provides raw data on the type, management and outcome of access complications of the entire cohort. The frequency of major / minor access complications was 9%; life-threatening bleeding occurred in 0.4% and mortality in 0.1% (i.e. 1 case of an arterial rupture). In Table 3**,** the data are summarized for the subgroup of TAVR procedures in which the Sapien S3 / Ultra or Evolut Pro-valve was used (i.e. the two most commonly used valves in contemporary practice). The frequency of major / minor access complications was 10% in the TAVR-group and none of the access complications in TAVR were associated with life-threatening bleeding or death. The main article demonstrated that the frequency of access complications in Roll-in cases (first or second time operator use of MANTA) was similar as compared to non-Roll-in cases (third time or more operator experience with MANTA). Table 4 demonstrates that this finding was despite the fact that Roll-in cases as a group had higher STS score as compared to patients not labelled as a Roll-in case (median STS score: 3.8 vs. 3.1%, respectively, *p* = 0.015). Fig. 1 demonstrates the MANTA VCD hemostasis times: 67% of patients had complete hemostasis within 1 min and 88% within 5 min. Because device profile determines arteriotomy size and complication risk, access complication frequencies were further stratified per valve-platform as shown in Fig. 2**.** It was found that the valve-platform exhibiting the smallest device profile (Evolut R) was associated with access complications in 7.1% while other (larger profile) valve-platforms were associated with complication rates between 8.3 and 13.1%.

**Table 1:** Study characteristics and in- and exclusion criteria.

	Mieghem et al. JACC Cardiovasc. Interv 2017^2^	Wood et al. Circ Cardiovasc. Interv 2019^3^	Kroon et al. Cath Cardiovasc. Interv. 2020^4^
Study name	CE Mark Study	SAFE Manta US Pivotal Study (PSD-19)	MARVEL
Design	Prospective, single arm, multicenter	Prospective, single arm, multicenter	Prospective, single arm, multicenter
Registration	NCT02521948 (study for CE mark approval)	G160115 (study for FDA approval)	NCT03330002 (Post market study)
Time period	Jul-2015–Jan-2016	Nov-2016–Sep 2017	Feb-2018–Jul 2019
Investigational sites^a^	3 in Europe	19 in United States, 1 in Canada	9 in Europe, 1 in Canada
No. of patients enrolled, total	50	341	500
No. of patients enrolled, Roll-in cases^b^	0	78	0
No. of operators^a^	9	42	31
Independent clinical event committee	yes	yes	yes
Data safety and monitoring	100% of data monitored by Factory-CRO (Bilthoven, the Netherlands)	100% event adjudication by Baim Institute for Clinical Research (Boston, MA); 100% of data monitored by Health Policy Associates Inc.	30% of data monitored by Factory-CRO (Bilthoven, the Netherlands)
Inclusion Criteria	Candidate for elective percutaneous interventional procedure with 12-F to 19F catheter size (sheath outer diameter 16-F to 24.5F)	Candidate for elective percutaneous interventional procedure with 10-F to 18-F catheter size	Candidate for elective percutaneous interventional procedure
		CFA diameter ≥5 mm for 14-F MANTA and ≥6 mm for 18-F Manta	
		Age ≥21 years	
Exclusion Criteria	Arterial puncture outside CFA	Significant anemia (Hb < 10 g/dL or Ht <30%)	Excessive calcification of the access vessel
	CFA size inappropriate for selected sheath size	Morbid obesity or cachexia (body mass index >40 kg/m^2^ or <20 kg/m^2^)	Severe periperal artery disease precluding safe introduction of a large arterial sheath
	Complicated CFA access (i.e. excessive hematoma surrounding puncture site, arteriovenous fistula, posterior wall puncture)	Known bleeding disorder	Marked tortuositu of the femoral or iliac artery
	Renal insufficiency (serum creatinine >2.5 mg/dl)	CFA excessive calcium precluding safe access in the opinion of the operator or severe peripheral vascular disease (on CT-A)	Morbid obesity or cachexia (body mass index >40 kg/m^2^ or <20 kg/m^2^)
	Inability to ambulate at baseline	Recent (<14 days) femoral artery puncture, incomplete healing of recent femoral artery puncture	Baseline systolic blood pressure >180 mmHg
		Left ventricular ejection fraction <20%	
		Renal insufficiency (serum creatinine >2.5 mg/dl) or on dialysis	
		Puncture site other than the CFA (i.e. profunda femoral artery, superficial femoral artery or at bifurcation of these arteries)	
		Marked tortuosity of femoral or iliac artery	
		Intraprocedural complications at femoral access site around the large bore sheath (i.e. angiographic evidence of thrombus or injury)	
		Activated clotting time > 250 s before removal of the sheath	
		Systolic blood pressure > 180 mmHg or diastolic >110 mmHg	

*Abbreviations:* CFA,common femoral artery; F, French; Hb, hemoglobin; Ht, hematocrit.

a Some investigational sites and operators participated in >1 study.

b Roll in cases were executed by operators with first or second time use of the MANTA vascular closure device, of which 78 cases stem from the SAFE MANTA study that were not included in the original trial.

**Table 2:** Data on access complications and management.

Case	Valve-platform (in case of TAVR)	Access complication details	Treatment	No. blood transfusions	Timing of complication (days after procedure)	Access complication (major or minor)	Bleeding complication (life-threatening/ disabling or major)
1	Sapien 3 / Ultra	stenosis	stent	0	0	major	no
2	Sapien 3 / Ultra	incomplete arteriotomy closure	compression	0	1	major	major
3	Evolut R	incomplete arteriotomy closure	Ethanol blood patch injection in inferior epigastric artery	3	6	major	major
4	Evolut PRO	occlusion	balloon	0	0	major	no
5	Sapien 3 / Ultra	stenosis	stent	0	0	major	no
6	Sapien 3 / Ultra	thrombotic occlusion	surgical repair	0	0	major	no
7	Evolut PRO	occlusion	surgical repair	0	0	major	no
8	Sapien 3 / Ultra	occlusion	balloon	0	0	major	major
9	*n.a.* (EVAR)	thrombotic occlusion	stent	0	0	major	no
10	Sapien 3 / Ultra	incomplete arteriotomy closure	stent	0	0	major	no
11	Sapien 3 / Ultra	thrombotic occlusion	balloon	0	0	major	no
12	Sapien 3 / Ultra	incomplete arteriotomy closure	compression	1	0	minor	major
13	Evolut R	incomplete arteriotomy closure	none	0	1	minor	major
14	Evolut R	incomplete arteriotomy closure	none	1	0	minor	major
15	Sapien 3 / Ultra	pseudoaneurysm	compression	0	0	minor	no
16	Sapien 3 / Ultra	pseudoaneurysm	none	0	1	minor	no
17	Sapien 3 / Ultra	incomplete arteriotomy closure	none	0	1	minor	no
18	Evolut R	pseudoaneurysm	compression	0	1	minor	no
19	Evolut PRO	nerve injury	none	0	0	minor	no
20	*n.a.* (EVAR)	pseudoaneurysm	none	0	27^a^	minor	no
21	*n.a.* (EVAR)	pseudoaneurysm	compression	0	1	minor	no
22	Sapien 3 / Ultra	pseudoaneurysm	none	0	57^a^	minor	no
23	*n.a.* (EVAR)	pseudoaneurysm	none	0	41^a^	minor	no
24	Sapien 3 / Ultra	thrombotic occlusion	balloon	0	0	major	no
25	Sapien 3 / Ultra	stenosis	none	1	0	major	no
26	Sapien 3 / Ultra	dissection	balloon	0	0	major	no
27	Sapien 3 / Ultra	occlusion	surgical repair	1	0	major	no
28	Sapien 3 / Ultra	occlusion	surgical repair	0	0	major	no
29	Sapien 3 / Ultra	occlusion	surgical repair	0	0	major	no
30	Sapien 3 / Ultra	stenosis	stent	0	0	major	no
31	Evolut R	pseudoaneurysm	compression	0	1	minor	no
32	*n.a.* (EVAR)	pseudoaneurysm	none	0	34^a^	minor	no
33	Sapien 3 / Ultra	thrombotic occlusion	surgical repair	0	0	major	major
34	Sapien 3 / Ultra	incomplete arteriotomy closure	surgical repair	2	0	major	major
35	Sapien 3 / Ultra	thrombotic occlusion	none	0	0	major	no
36	Evolut PRO	incomplete arteriotomy closure	surgical repair	2	0	major	no
37	Evolut PRO	pseudoaneurysm	stent	2	0	major	major
38	Evolut PRO	incomplete arteriotomy closure	none	0	0	major	no
39	Accurate Neo	pseudoaneurysm	balloon	2	0	major	major
40	*n.a.* (EVAR)	incomplete arteriotomy closure	surgical repair	0	0	major	major
41	Evolut PRO	incomplete arteriotomy closure	surgical repair	2	0	major	no
42	Evolut R	incomplete arteriotomy closure	stent	4	0	major	life-threatening/disabling
43	Evolut PRO	dissection	surgical repair	0	0	major	no
44	Evolut PRO	dissection	surgical repair	0	0	major	no
45	Sapien 3 / Ultra	thrombotic occlusion	surgical repair	0	0	major	no
46	*n.a.* (aortic valvuloplasty)	incomplete arteriotomy closure	surgical repair	2	0	major	life-threatening/disabling^b^
47	Sapien 3 / Ultra	incomplete arteriotomy closure	balloon	2	0	major	major
48	Accurate Neo	stenosis	surgical repair	3	0	major	life-threatening/disabling
49	Evolut R	incomplete arteriotomy closure	surgical repair	8	0	major	life-threatening/disabling
50	Accurate Neo	incomplete arteriotomy closure	surgical repair	3	0	major	major
51	Evolut PRO	incomplete arteriotomy closure	compression	0	0	major	major
52	Sapien 3 / Ultra	stenosis	stent	0	0	major	no
53	Sapien 3 / Ultra	dissection	compression	0	0	minor	no
54	Evolut PRO	dissection	balloon	0	0	minor	no
55	Evolut PRO	dissection	stent	0	0	minor	no
56	Evolut PRO	dissection	none	0	0	minor	no
57	Sapien 3 / Ultra	dissection	none	0	0	minor	no
58	Sapien 3 / Ultra	dissection	compression	0	0	minor	no
59	Evolut PRO	dissection	none	0	0	minor	no
60	Evolut PRO	dissection	stent	0	0	minor	no
61	Accurate Neo	dissection	stent	0	0	minor	no
62	Sapien 3 / Ultra	stenosis	compression	0	0	minor	no
63	Sapien 3 / Ultra	stenosis	balloon	0	0	minor	no
64	Evolut PRO	stenosis	surgical repair	0	0	minor	no
65	Evolut PRO	pseudoaneurysm	none	0	1	minor	no
66	Sapien 3 / Ultra	pseudoaneurysm	none	0	0	minor	no
67	Evolut R	pseudoaneurysm	compression	0	0	minor	no
68	Evolut R	pseudoaneurysm	lidocaine/epinephrine combination or thrombin injection	0	0	minor	no
69	Sapien 3 / Ultra	pseudoaneurysm	lidocaine/epinephrine combination or thrombin injection	1	1	minor	no
70	Accurate Neo	pseudoaneurysm	balloon	0	0	minor	no
71	Accurate Neo	pseudoaneurysm	balloon	0	0	minor	no
72	Sapien 3 / Ultra	incomplete arteriotomy closure	compression	0	0	minor	no
73	Sapien 3 / Ultra	incomplete arteriotomy closure	lidocaine/epinephrine combination or thrombin injection	0	0	minor	no
74	Sapien 3 / Ultra	incomplete arteriotomy closure	lidocaine/epinephrine combination or thrombin injection	0	0	minor	no
75	Sapien 3 / Ultra	incomplete arteriotomy closure	lidocaine/epinephrine combination or thrombin injection	0	0	minor	no
76	Sapien 3 / Ultra	incomplete arteriotomy closure	compression	0	1	minor	no
77	Sapien 3 / Ultra	incomplete arteriotomy closure	surgical repair	0	0	minor	no
78	Sapien 3 / Ultra	incomplete arteriotomy closure	stent	0	0	minor	no
79	Sapien 3 / Ultra	incomplete arteriotomy closure	none	0	0	minor	no
80	Evolut PRO	incomplete arteriotomy closure	balloon	0	0	minor	no
81	Sapien 3 / Ultra	incomplete arteriotomy closure	surgical repair	0	0	major	major

*Abbreviations:* EVAR, endovascular aortic repair; TAVR, transcatheter aortic valve replacement.

a Complication diagnosed after discharge from primary hospital admission.

b Complication leading to death.

**Table 3:** Subgroup analysis of access complications and management in patients undergoing TAVR with SapienS3 / Ultra or Evolut PRO valves.

	Access complications		
	minor	major	all
	*n* = 29 (5.0%)^a^	*n* = 30 (5.2%)^a^	*n* = 59 (10.2%)^a^
**Type of vascular injury**			
Incomplete arteriotomy closure	11 (1.9)	9 (1.6)	20 (3.4)
Dissection	8 (1.4)	3 (0.5)	11 (1.9)
Stenosis	3 (0.5)	5 (0.9)	8 (1.4)
Occlusion	0	12 (2.1)	12 (2.1)
Pseudo-aneurysm	6 (1.0)	1 (0.2)	7 (1.2)
Transient nerve injury	1 (0.2)	0	1 (0.2)
**Treatment**			
Surgical repair	2 (0.3)	13 (2.2)	15 (2.6)
Stenting	3 (0.5)	6 (1.0)	9 (1.6)
Prolonged balloon inflation	3 (0.5)	6 (0.8)	9 (1.6)
None / manual compression	17 (2.9)	5 (0.7)	22 (3.8)
Percutaneous injection^b^	4 (0.7)	0	4 (0.7)
**Bleeding complications**			
Life-threatening or disabling	0	0	0
Major	0	9 (1.6)	9 (1.6)

a Data are presented as n (%, out of a total of 580 patients treated with Edwards Sapien S3 / Ultra or Evolut PRO valves).

b All patients underwent thrombin or lidocaine injection, except one patient who onderwent ethanol injection in the inferior epigastric artery.

**Table 4:** Baseline and peri‑procedural characteristics stratified according to Roll-in case.

	Total	No Roll-in case^a^	Roll-in case^a^	
Characteristic	*N* = 891	*N* = 813	*N* = 78	p-value
**Baseline charactistics**				
Age, mean (SD), y	80 (8)	80 (7)	78 (10)	0.004
Female gender	364 (41)	346 (43)	18 (23)	0.001
Body mass index, median (IQR), kg/m^2^	27 (24–30)	27 (24–30)	28 (25–32)	0.057
Peripheral vascular disease	91 (10)	76 (9)	15 (19)	0.006
Previous coronary artery bypass graft	126 (14)	104 (13)	22 (28)	<0.001
Previous percutaneous coronary intervention	263 (30)	239 (29)	24 (31)	0.80
Previous cerebrovascular event	94 (11)	94 (12)	0	<0.001
Permanent pacemaker	87 (10)	75 (9)	12 (15)	0.080
Glomerular filtration rate < 60 mL/min	453 (51)	409 (50)	44 (56)	0.31
Society of Thoracic Surgeons' score, median (IQR), %	3.2 (2.1–4.9)	3.1 (2.1–4.7)	3.8 (2.5–5.5)	0.015
Oral anticoagulant	199 (22)	190 (23)	9 (12)	0.017
New oral anticoagulant	87 (10)	84 (10)	3 (4)	0.072
**Procedural characteristics**				
Activated clotting time before closure, median (IQR), sec	175 (142–217)	172 (142–218)	190 (156–213)	0.23
Systolic blood pressure before closure, mean (SD), mmHg	132 (23)	132 (23)	124 (20)	0.001
Protamine used before closure	592 (66)	531 (65)	61 (78)	0.021
Procedure duration, median (IQR), min	65 (48–87)	64 (46–85)	75 (56–101)	0.004
Time to haemostasis, median (IQR), sec	31 (17–76)	32 (17–83)	27 (20–45)	0.55
**Post Procedural characteristics**				
Length of stay, median (IQR), days	2 (1–5)	3 (2–5)	2 (1–2)	<0.001

a Roll-in case indicates an operator first or second time use of the MANTA vascular closure device. Roll-in cases were excluded in the Device Exemption Primary Analysis Cohort of the SAFE MANTA study.

## 2 Experimental Design, Materials and Methods

As mentioned above, this patient-level meta-analysis pooled data from two medical device approval studies and one post-approval registry to assess the frequency, impact and predictors of MANTA related access complications after large-bore catheter-based cardiovascular interventions. Procedures were performed by 71 operators at 28 sites between 2015 and 2019. Table 1 describes all in- and exclusion criteria of each of the 3 studies from which data were derived. Overall, patients were eligible if they underwent percutaneous cardiovascular interventions and planned access closure using the MANTA VCD. Exclusion criteria were most stringent in the SAFE MANTA trial, followed by the CE mark study whereas MARVEL applied liberal and only relative exclusion criteria. The most important exclusion criteria were morbid obesity or cachexia (body mass index >40 or <20 kg/m^2^), excessive femoral calcium or severe peripheral vascular disease, marked tortuosity of the iliofemoral tract and puncture site other than the common femoral artery. Of note, in SAFE MANTA poor left ventricular function and severe renal dysfunction were also exclusion criteria. In all patients, major and minor access complications were defined according to the updated Valve Academic Research Consortium 2 criteria [5]. All events were adjudicated by independent clinical event committees. A detailed description of the study population, MANTA device, the percutaneous procedures, ileofemoral data and clinical outcome assessment is presented in the main article [1]. Continuous variables were compared using the Student t-test or Mann Whitney U test when appropriate. Categorical variables are presented as numbers and percentages of patients and categorical variables were compared with the Chi square test. A two-sided *p*<0.05 was considered to indicate significance. Statistical analyses were performed using Statistical Package for the Social Sciences version 25 (IBM, Armonk, New York)

## Ethics Statement

Informed consent was obtained from all patients that were enrolled with the use of a prespecified patient information form. The herein reported data were derived from the SAFE-MANTA study (protocol identifier: PSD-109), CE-Mark study (protocol identifier: PSD-051) and MARVEL registry (protocol identifier: PSD-212), and study protocols were approved by the Ethics Committees of each participating center.

## CRediT Author Statement

**Rutger-Jan Nuis:** Conceptualization, Methodology, Formal analysis, Investigation, Data Curation, Visualization, Writing – Original Draft; **David Wood:** Conceptualization, Writing - Review & Editing; **Herbert Kroon:** Writing - Review & Editing; **Maarten van Wiechen:** Writing - Review & Editing; **Darra Bigelow:** Writing - Review & Editing; **Chris Buller:** Writing - Review & Editing; **Joost Daemen:** Writing - Review & Editing; **Peter de Jaegere:** Writing - Review & Editing; **Zvonimir Krajcer:** Writing - Review & Editing; **John Webb:** Writing - Review & Editing; **Nicolas Van Mieghem:** Conceptualization, Methodology, Writing - Review & Editing.

## Declaration of Competing Interest

Dr. Van Mieghem reports receiving grant support from Teleflex. The other authors declare that they have no known competing financial interests or personal relationships which have or could be perceived to have influenced the work reported in this article.
